# Association between impaired glucose metabolism and long-term prognosis at the time of diagnosis of depression: Impaired glucose metabolism as a promising biomarker proposed through a machine-learning approach

**DOI:** 10.1192/j.eurpsy.2023.10

**Published:** 2023-02-03

**Authors:** Dong Yun Lee, Yong Hyuk Cho, Myoungsuk Kim, Chang-Won Jeong, Jae Myung Cha, Geun Hui Won, Jai Sung Noh, Sang Joon Son, Rae Woong Park

**Affiliations:** 1 Department of Biomedical Informatics, Ajou University School of Medicine, Suwon, Korea; 2 Department of Psychiatry, Ajou University School of Medicine, Suwon, Korea; 3 Data Science Team, Evidnet Co Ltd, Pangyo, Korea; 4 Medical Convergence Research Center, Wonkwang University, Iksan, Korea; 5 Department of Gastroenterology, Gang Dong Kyung Hee University Hospital, Seoul, Korea; 6 Department of Psychiatry, Catholic University of Daegu School of Medicine, Daegu, Korea; 7 Department of Medical Sciences, Graduate School of Ajou University, Suwon, South Korea

**Keywords:** Biomarker, depression, impaired glucose metabolism, long-term prognosis, machine learning

## Abstract

**Background:**

Predicting the course of depression is necessary for personalized treatment. Impaired glucose metabolism (IGM) was introduced as a promising depression biomarker, but no consensus was made. This study aimed to predict IGM at the time of depression diagnosis and examine the relationship between long-term prognosis and predicted results.

**Methods:**

Clinical data were extracted from four electronic health records in South Korea. The study population included patients with depression, and the outcome was IGM within 1 year. One database was used to develop the model using three algorithms. External validation was performed using the best algorithm across the three databases. The area under the curve (AUC) was calculated to determine the model’s performance. Kaplan–Meier and Cox survival analyses of the risk of hospitalization for depression as the long-term outcome were performed. A meta-analysis of the long-term outcome was performed across the four databases.

**Results:**

A prediction model was developed using the data of 3,668 people, with an AUC of 0.781 with least absolute shrinkage and selection operator (LASSO) logistic regression. In the external validation, the AUCs were 0.643, 0.610, and 0.515. Through the predicted results, survival analysis and meta-analysis were performed; the hazard ratios of risk of hospitalization for depression in patients predicted to have IGM was 1.20 (95% confidence interval [CI] 1.02–1.41, *p* = 0.027) at a 3-year follow-up.

**Conclusions:**

We developed prediction models for IGM occurrence within a year. The predicted results were related to the long-term prognosis of depression, presenting as a promising IGM biomarker related to the prognosis of depression.

## Introduction

Depression severely restricts individual psychosocial functions and lowers the quality of life. It leads to national problems such as increased suicide rates and medical expenses because of its chronicity. The World Health Organization cited major depressive disorder as the third cause of the global burden of disease in 2008 and predicted that depression would rank first by 2030 [1]. Numerous factors such as biological markers and poor habits [2] are linked to the onset and recovery of depression. Variable clinical patterns, unpredictable progression and prognosis, and insufficient therapeutic response make depression treatment challenging for clinicians. Remission rates with antidepressants are also overall low (~27% as per the STAR*D trial) [3], and 20%–25% of patients with depression are at risk of chronic depression [4]. Thus, previous studies have tried to improve treatment outcomes of depression, and evidence has revealed that early intervention of depression is not only associated with better treatment response and long-term outcomes but also with slow disease progression [5–8]. These studies gradually focused on exploring various variables that can predict prognosis in the early stages of depression and achieving personalized treatment through targeted treatment strategies [9–11].

Previous studies have suggested that measuring metabolic markers may be a promising way of predicting long-term clinical outcomes in depression [12]. Recent studies have revealed the relationship among depression, suicidal behavior, insulin resistance (IR), or impaired glucose metabolism (IGM), and evidence of their interactions is accumulating [13–16]. Specifically, IGM is characterized by glucose metabolic disturbance and is defined as prediabetes mellitus (DM) and DM [17]. It can be measured based on hemoglobin A1C (HbA1c) levels in the blood and fasting blood sugar; thus, its clinical utility is high. Many studies have reported that IGM has a bidirectional association with depression. A study reported that reduced serotonin levels were associated with elevated blood glucose levels, Insulin Resistance (IR), and depressed mood [18]. Some previous studies have found that higher glucose levels are associated with dysthymia and higher HbA1c concentrations with recurrent or psychotic depression [19]. In addition, a study in adults with type 2 DM (T2DM) found that certain antidiabetic drugs were associated with a lower risk of depression [20]. Recently, a cross-sectional study correlated IR with depression severity as an endophenotype of depression [13]. Despite these studies, no consensus has yet been reached to the extent that the association between IGM and depression is applicable to clinical practice for establishing patient care strategies.

Machine-learning (ML)-based predictive models are becoming increasingly popular by combining huge data into one model. For depression, conventional regression methods have limitations in prediction; not only well-known demographic factors or factors related to typical treatment but also various comorbidity with physical disease and generally polypharmacy are common [19, 21]. By contrast, ML-based methods have successfully predicted depression persistence, chronicity, severity [22], treatment response, and first and new onset of depressive episodes [17, 18, 23].

This study aimed to investigate whether IGM could be utilized as a biomarker that reflects the clinical severity and prognosis of depression. Initially, we attempted to develop a model that predicts IGM occurrence at the time of the first diagnosis of depression through an ML algorithm. Subsequently, using multicenter and longitudinal data, we intended to analyze and validate whether the IGM occurrence predicted by the model is related to the short-term and long-term prognosis of depression.

## Methods

### Data source

This study used data from approximately 6 million patients across the four electronic health record databases in South Korea: Ajou University School of Medicine (AUSOM), Daegu Catholic Medical Center (DCMC), Wonkwang University Hospital (WKUH), and Kyung Hee University Hospital at Gangdong (KHNMC) (Supplementary Material S1). The clinical data included diagnoses, observations, provider visits, procedures performed, and medications filled. The databases were formatted according to the Observational Medical Outcomes Partnership–Common Data Model version 5.3.1, maintained by the Observational Health Data Sciences and Informatics (OHDSI), and de-identified [24]. The database of AUSOM was used in model development, and the other three databases were used to validate the developed model. After the development and validation of the model, all databases were used in the survival analysis.

This study was approved by the Institutional Review Board of the Ajou University Hospital (AJOUIRB-MDB-2022-255). Informed consent was not required owing to the use of de-identified data. Access to DCMC, WKUH, and KHNMC databases during the external validation process was allowed under the IRB mutual recognition agreement (research-free zone agreement).

### Study population and outcome

The study population included patients with a new depressive episode. The index date was defined as the patient’s first diagnosis of depressive disorder. To verify their first diagnosis of depressive disorder, at least 1 year of observation before the index date was required. Within the 1-year observation period before the index date, relevant covariates on each patient were collected to predict their future diagnosis of IGM. Patients who were treated for depression, those who had antidepressant prescriptions, and had undergone psychiatric procedures after the index date were included. Also, patients who had at least 1 year of follow-up after the index date were included. For the IGM prediction, patients who had at least one measure of HbA1c or fasting glucose within 1 year after the index date were included. As exclusion criteria, patients with diagnosis of bipolar disorder, schizophrenia, and psychosis on or before the index date were excluded. Regarding DM, a previous history of DM, DM complications, and exposures to antidiabetic drugs were excluded.

The primary outcome for the predictive models was IGM within 1 year after the index date. IGM was defined as pre-DM or T2DM and measured by HbA1c or fasting glucose. For IGM, HbA1c levels were defined as ≥5.6%, and fasting plasma glucose as ≥100 mg/dL [25]. All patients with depression were followed up for 1 year. If IGM occurred within this 1-year period, the observation was stopped on the day that the IGM diagnosis was coded. Thus, the predictive models were developed using the primary outcome. After that, patients were divided into “predicted to have IGM” and “predicted not to have IGM” groups through a predictive model at the time of the index date. Further details of the cohort definitions and code lists are presented in Supplementary Materials S2–S3.

### Model development

We used the patient-level prediction framework of the OHDSI to develop and validate the predictive models. This framework consisted of standardized model development and validation processes that require defining predictable problems and selecting the study population, outcomes, population settings, predictors, and statistical algorithms [26]. The predictive variables for model training were extracted and dichotomized for existence within short-term (30 days) and long-term (365 days) intervals before the index. The variables included patient age, sex, month of the index visit, diagnoses, drug exposures, and procedures. Through this process, 22,904 candidate variables were generated. The models were developed across multiple algorithms, including least absolute shrinkage and selection operator (LASSO)-penalized regression, random forest, and extreme gradient boosting (XGBoost) via threefold cross-validation. The algorithm with the best performance was selected for the final model according to the value of the area under the receiver operating characteristic curve (AUROC).

### External validation

External validation was conducted to confirm the validity of the model’s performance using the databases of DCMC, WKUH, and KHNMC. Specifically, we evaluated the performance of the final model to other databases in the same setting as in the model development.

### Follow-up and long-term outcome measurements

The patients were followed up 3 years after the index date. During the follow-up, risk of hospitalization for depression in patients who were predicted to have IGM compared with patients who were predicted not to have IGM. Risk of hospitalization for depression was defined as hospitalization caused by the exacerbation of depressive episodes. In addition, rehospitalization after discharge for the first diagnosis was considered [27]. To distinguish between existing hospitalization and rehospitalization, only hospitalization after at least a 2-week washout period was defined as an outcome. The outcomes were binarized into hospitalization and non-hospitalization based on the occurrences recorded in the databases.

### Statistical analysis

Descriptive statistical analyses were appropriately performed. Baseline characteristics are presented as counts with proportions for categorical variables and as median with interquartile range for continuous variables. The chi-square test was used to compare categorical variables between populations. Accuracy, AUROC, and area under the precision and recall curve (AUPRC) were calculated to evaluate the performance of the prediction models. We used the maximal Youden index to select the optimal cutoff value in the prediction model [28].

Moreover, we verified whether the group predicted by the final model was related to the actual IGM occurrence. The final model was used to estimate their predicted IGM at the internal validation dataset, and patients with a relatively high probability of IGM were then labeled as predicted to have IGM. If the patients in the internal validation dataset were predicted to have IGM, they were classified as “predicted to have IGM,” and others were classified as “predicted not to have IGM.” The Kaplan–Meier survival analysis and log-rank test were used to analyze the difference in the occurrence of IGM within 1 year after the index date in the group predicted to have IGM versus the group predicted not to have IGM.

After model development and external validation, Kaplan–Meier and Cox survival analyses for the long-term outcomes were performed to assess the risk of hospitalization for depression in patients who have IGM, as determined by the final model. Then, a meta-analysis was performed to calculate the summary hazard ratio (HR) estimates across four databases.

All *p*-values <0.05 were considered statistically significant. All analyses were conducted using R software version 3.6 (R Foundation for Statistical Computing, Vienna, Austria), OHDSI’s Health Analytics Data to Evidence Suite packages, and open-source statistical R packages.

## Results

### Baseline characteristics

A total of 481 outcomes in 3,668 patients from AUSOM were used for model development, and for the external validation, 543 outcomes in a total of 5,716 patients (DCMC, *n =* 2,129; WKUH, *n =* 2,717; and KHNMC, *n =* 870) were used. Table 1 shows the baseline characteristics of the study population in AUSOM. The baseline characteristics of other databases are presented in Supplementary Tables S1–S3. Among the 3,668 patients with depression in the AUSOM database, 481 (13.1%) experienced IGM within 1 year after the diagnosis of depression. No significant differences were found in age, sex, medical history except hypertension, and psychiatric history between the groups. The proportion of hypertension was significantly lower in with IGM group (*p* < 0.01). Middle-aged (40–59 years) and female patients were the most predominant in the study population. Hypertension and anxiety disorder were frequent diagnoses (hypertension, 15.2% and 9.1%; anxiety disorder, 15.4% and 14.1%, respectively).

**Table 1.** Baseline characteristics for study population with or without IGM in AUSOM.

Variable	Without IGM (*n* = 3,187)	With IGM (*n* = 481)	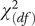	*p*-value
Age group, *n* (%)			0.34(3)	0.95
<20	217 (6.8)	31 (6.4)		
20–39	629 (19.7)	109 (22.7)		
40–59	1,355 (42.5)	205 (42.6)		
≥60	986 (30.9)	136 (28.3)		
Sex, *n* (%)				
Male	947 (29.7)	135 (28.1)	0.47(1)	0.49
Medical history, *n* (%)				
Chronic liver disease	43 (1.3)	2 (0.4)	2.28(1)	0.13
Renal impairment	48 (1.5)	4 (0.8)	0.92(1)	0.33
Hyperlipidemia	83 (2.6)	5 (1.4)	3.72(1)	0.05
Obesity	63 (1.9)	10 (2.1)	0.01(1)	1.00
Hypertension	487 (15.2)	44 (9.1)	12.21(1)	<0.01*
Rheumatoid arthritis	32 (1.0)	2 (0.5)	0.99(1)	0.31
Psychiatric history, *n* (%)				
Anxiety disorder	492 (15.4)	68 (14.1)	0.45(1)	0.50
Sleep disorder	310 (9.7)	33 (6.8)	3.72(1)	0.05
Neurodevelopmental disorder	55 (1.7)	7 (1.5)	0.05(1)	0.81

*Note*: 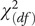 , chi-square value and degree of freedom.

Abbreviations: AUSOM, Ajou university school of medicine; IGM, impaired glucose metabolism.

* indicates statistical significance (*p* < 0.05).

### Prediction models

**Figure 1.**
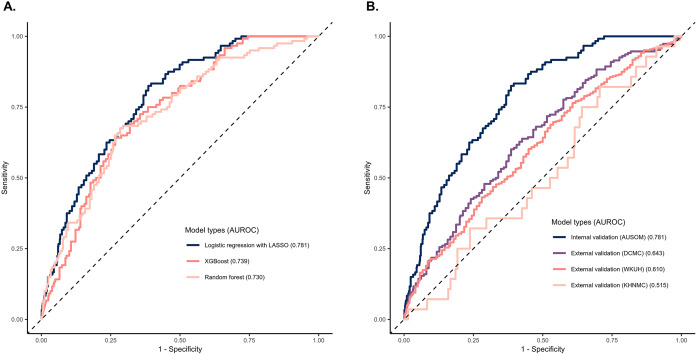
Receiver operating characteristic (ROC) curve of models predicting impaired glucose metabolism. (A) ROC curve for the models according to algorithms. (B) ROC curve for internal and external validations. The performance of the models using the area under the receiver operating characteristic curve is compared.

Figure 1 shows the performance of the ML model in the internal validation set of AUSOM, including LASSO, random forest, and XGBoost. The best-performing model, selected by comparing the average AUROC from the threefold validation, was a logistic regression with LASSO. We defined LASSO as the final model, which showed an AUROC of 0.781 (95% CI 0.742–0.820) on the internal validation dataset. The accuracy and AUPRC of the final model were 0.667 and 0.338, respectively. The performance metrics are shown in Supplementary Table S4.

Table 2 shows the top 10 important predictors. The feature importance analysis showed that a normal range of blood glucose levels before depression diagnosis was the most important predictor across the three algorithms. Drug exposures such as antipsychotics were important predictors in the prediction models. Three models consistently considered the category of the blood test as important predictors. Unlike other models, the LR with LASSO model included the category of image test as a predictor. In Supplementary Table S5, the predictors that increase the IGM prediction risk and those that decrease the risk are indicated in red and blue, respectively.

**Table 2.** Top 10 important predictors of the prediction models for impaired glucose metabolism.

Rank	LR with LASSO	XGBoost	Random forest
1	Normal range of blood glucose level within 1 year before diagnosis	Normal range of blood glucose level within 1 year before diagnosis	Normal range of blood glucose level within 1 year before diagnosis
2	Normal range of blood calcium level within 1 year before diagnosis	Measurement of blood aspartate aminotransferase level within 1 year before diagnosis	Measurement of blood alanine aminotransferase level within 1 year before diagnosis
3	Measurement of urinalysis within 1 month before diagnosis	Measurement of blood triglyceride within 1 year before diagnosis	Measurement of blood cholesterol level within 1 year before diagnosis
4	Measurement of blood platelet within 1 year before diagnosis	NSAIDs prescription within 1 month before diagnosis	Measurement of blood alanine aminotransferase level within 1 year before diagnosis
5	CT within 1 month before diagnosis	Measurement of urinalysis within 1 year before diagnosis	Measurement of blood aspartate aminotransferase level within 1 year before diagnosis
6	Measurement of C reactive protein within 1 month before diagnosis	Antipsychotics prescription within 1 year before diagnosis	Normal range of blood bilirubin level within 1 year before diagnosis
7	Drugs for peptic ulcer prescription at diagnosis	SSRI prescription at diagnosis	Measurement of uric acid level within 1 year before diagnosis
8	Measurement of blood cholesterol level within 1 year before diagnosis	HMG CoA reductase inhibitors prescription within 1 month before diagnosis	Measurement of creatine level within 1 year before diagnosis
9	Antipsychotics prescription within 1 year before diagnosis	Measurement of blood hepatitis B virus test within 1 month before diagnosis	Measurement of blood albumin level within 1 year before diagnosis
10	Normal range of blood aspartate aminotransferase level within 1 year before diagnosis	Haloperidol prescription at diagnosis	Measurement of blood protein level within 1 year before diagnosis

*Note*: The color in the table means the category of features (orange: laboratory test, green: image test, and blue: drug exposure).

Abbreviations: CT, computed tomography; LASSO, least absolute shrinkage and selection operator; LR, logistic regression; NSAID, non-steroidal anti-inflammatory drugs; SSRI, selective serotonin reuptake inhibitor; XGBoost, extreme gradient boosting.

### External model validation

The final model was externally validated using the DCMC, WKUH, and KHNMC databases. In the external validation databases, patients experienced IGM at a rate of 8.8% (188/2,129) in DCMC, 12.0% (327/2,717) in WKUH, and 3.2% (28/870) in KHNMC. The external validation performance of the final model regarding AUROC was 0.643 at DCMC, 0.610 at WKUH, and 0.515 at KHNMC.

### Long-term outcomes of ML-predicted IGM

**Figure 2.**
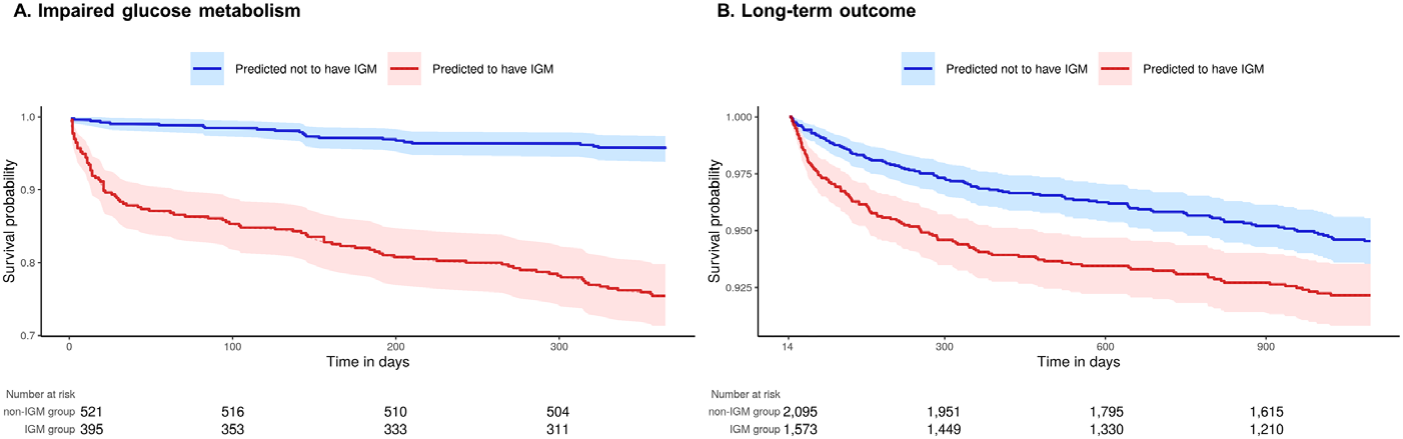
Kaplan–Meier curves in the stratified survival analysis. (A) Impaired glucose metabolism in the internal validation dataset of AUSOM. (B) Long-term outcome for the 3-year follow-up in the overall cohort of AUSOM.

Figure 2 shows the clinical benefit of using the IGM prediction models. In the internal validation dataset of AUSOM, the group predicted to have IGM had a significantly higher occurrence of IGM within 1 year after the index date than the group predicted not to have IGM (log rank, *p* < 0.001). Furthermore, patients predicted to have IGM showed significantly worse long-term outcomes. In the overall cohort of AUSOM, survival analysis showed that the risk of hospitalization for depression occurred more frequently in patients who were predicted to have IGM during the 3-year follow-up (log rank, *p* = 0.002) (Figure 2).

**Figure 3.**
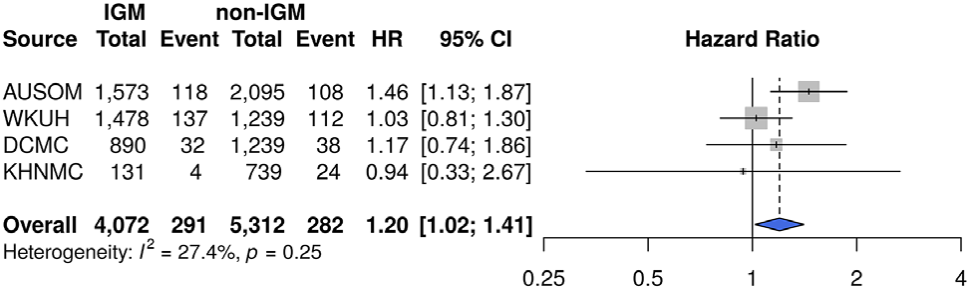
Risk of long-term outcome in 3 years in patients predicted by the machine-learning model to have IGM within 1 year.

We further assessed long-term outcomes not only in AUSOM but also in external validation databases. The meta-analytic comparative effect estimates for the risk of hospitalization for depression are presented in Figure 3. The summary HR of risk of hospitalization for depression during the 3-year follow-up was 1.20 (95% CI 1.02–1.41, *p* = 0.027) for patients predicted to have IGM.

## Discussion

We constructed a model to predict the occurrence of IGM within 1 year at the time of depression diagnosis using ML algorithms. By analyzing the longitudinal data of multiple institutions using this prediction model, we identified relationships between IGM prediction and the long-term prognosis of depression. Thus, IGM might be a promising biomarker associated with the prognosis of depression.

Despite being a common psychiatric disease, depression has a low treatment success rate because of the heterogeneity and difficulty in predicting its course [3, 29]. Thus, clinicians desire to identify biomarkers that can reflect the severity or chronicity of depression. Several previous studies have shown a complex relationship between depression and IGM. Knol et al. [30] reported in a meta-analysis a 37% increased risk of T2DM development in adults with depression compared with individuals without depression. Several possibilities have been suggested, and there are reports that hypothalamic–pituitary–adrenal axis abnormalities in patients with depression, hypercortisolemia, and immune system abnormalities, including chronic low-grade inflammations, influence the insulin effect [5, 6, 18]. Conversely, IGM including DM is related to the development or exacerbation of depression and the reactivity of antidepressants [31, 32]. The dysfunction of insulin receptors and subsequent signal cascade, which are related to IGM, has a direct effect on neural metabolism and the brain and is associated with depression by causing abnormalities in neurotransmitter metabolisms such as dopamine, serotonin, and norepinephrine [33, 34]. Moreover, some studies have revealed that the successful treatment of depression can correct insulin response, particularly with more serotonergic agents, such as selective serotonin reuptake inhibitors (SSRIs) [35, 36]. However, a recent study reported that low doses of metformin, DPP4 inhibitors, GLP1 analogs, and especially SGLT2 inhibitors were associated with lower odds of depression than non-users of these medications [20]. In summary, bidirectional pathophysiological connections exist between depression and IGM. This connection means that depression and IGM are important factors not only in each other’s pathogenesis but also in each other’s successful treatment and prognosis.

Recently, a nationwide study revealed that glucose disturbance is associated with increased suicidal ideation and suicidal behavior in patients with depression [37]. In another large-scale study, IR was proposed as a promising marker that reflects severity and chronicity in patients with depression [13]. These large-scale cross-sectional studies opened with a prelude to the relationship between IR and depression. Consequently, clinicians are paying attention to predicting IGM including IR in the early stages of diagnosis, and various treatment strategies can be implemented considering the long-term prognosis and treatment reactivity of patients with depression. However, depression and IGM have a complex relationship, and predicting IGM in the early stages of depression is not easy; thus, analysis using large-scale variables is needed because conventional analysis has limitations.

Data-driven ML algorithms are in the spotlight as a breakthrough in the discovery of hidden predictors and known clinically meaningful predictors selected by researchers [10]. Therefore, in this study, an IGM prediction model was developed using a data-driven ML algorithm. Specifically, the data used in this study consisted of a large number of tabular data, which was advantageous for the use of ML algorithms such as XGBoost, LR with LASSO, and random forest, similar to previous studies [38]. The model using LR with LASSO showed the highest performance in this study (Figure 1).

Since this study developed an IGM prediction model through an ML algorithm rather than deep learning, understandable explanations for prediction were obtained. Initially, at the time of diagnosis of depression, antipsychotics, including haloperidol, are commonly prescribed. Moreover, studies have reported that antipsychotics are related to an increase in blood sugar [39]. In addition, several studies have reported that benzodiazepine [40], corticosteroid [41], and peptic ulcer prescription, which are expected to be proton pump inhibitors [42], are associated with increased blood sugar and DM. CT, C reactive protein measurement, hepatitis B virus test, uric acid measurement, and nonsteroidal anti-inflammatory drug prescriptions are also observed as important predictors. They may have been tested for certain symptoms or prescribed drugs as an extension of the immune system’s dysfunction observed in depression and IGM. Ken et al. revealed that chronic low-grade inflammatory reactions in depression lead to apoptosis in pancreatic beta cells, which is related to IGM [43]. The increase in cytokine levels in patients with depression is linked to metabolic disturbance [34]. We suggest the immunological vulnerability in patients at the time of diagnosis of depression was reflected as a predictor. On the contrary, the most important predictor is “normal range of blood glucose level within 1 year before diagnosis” in all algorithms. Thus, if the blood glucose level within 1 year at the time of diagnosis of depression was normal, this time is not enough to observe the progression to IGM within 1 year. Measuring the levels of cholesterol, triglyceride, urinalysis, creatinine, etc., through blood tests also had a negative relationship with predicting IGM. This can be interpreted in the same way as the results of previous studies that continuity of care had some benefits including prevention of chronic diseases including DM [44]. Finally, unlike other drugs, SSRIs showed a negative relationship with predicting IGM. Although this is controversial, SSRIs had a positive effect on blood sugar control among antidepressants [45, 46].

Furthermore, in this study, survival analysis was conducted to determine whether the results of the IGM incidence prediction model were related to the 3-year prognoses of depression. Through a meta-analysis using four other hospital data with the Common Data Model (CDM) database, we identified differences in hospitalization caused by exacerbation of depressive episodes between the two groups divided into IGM prediction models using longitudinal data. This result can be interpreted through the report of previous studies that when depression or anxiety is accompanied by DM, disease burden and emotional distress increase because of poor metabolic control, low rates of blood glucose self-monitoring, and DM complications, which can predict inadequate response to depression treatment [36, 47].

This study has several limitations. First, this study used data from Koreans only; thus, the results cannot be generalized. However, this study showed that the CDM developed through a distributed research network enables a more efficient meta-analysis than in the past without exposing private information. This suggests that a global meta-analysis is possible if the same CDM is established in various countries. Second, this study used longitudinal data, but it has the limitations of a retrospective study. To clarify the relationship between depression and IGM, a prospective study is required. Third, this study did not include social and environmental factors that would be related to depression and IGM in the model development. This is also a limitation of the psychiatric CDM. Thus, developing measurable environmental and sociological variables is necessary. Fourth, model performance was reduced in the external validation of the IGM prediction model. Model performance commonly decreases in the external validation because of the varying characteristics of the enrolled participants, and it is difficult to control them all. Specifically, the external validation performance was low in the analysis of KHNMC compared with AUSOM. The result was assumed to be caused by the varying rates of IGM occurrence, i.e., 13.1% in AUSOM and 3.2% in KHNMC. Furthermore, since there is no overall difference between patients with and without IGM in the baseline characteristics, predicting IGM may be difficult. Fifth, indirect indicators such as depression-related hospitalization were used to determine the relationship between the results of the IGM prediction model and the long-term prognosis of depression. However, several recent studies have derived meaningful results using operational definitions such as this study [27]. Sixth, we included only individuals who were assessed for IGM for the study population. Among patients with depression in the study database, those with IGM assessment had a higher rate of comorbidities than those without IGM assessment. This suggests that the generalization of the results should be cautious.

In summary, we developed an IGM prediction model at the time of depression diagnosis using an ML algorithm and found a relationship between the results of the IGM prediction model and the long-term prognosis of depression using longitudinal data. Thus, we suggest that IGM is likely to be a promising biomarker in predicting the prognosis of depression. Treatment strategies should be established to improve metabolic disturbance, including IGM, and the use of IGM as an evaluation index for lifestyle modification and increased treatment success rate may be expected. Therefore, a more customized and multidimensional approach to the evaluation and treatment of depression would be possible.

## Data Availability

CDM data are designed to support a distributed research network. Thus, access to the data is restricted on internal private networks. Therefore, data are not publicly available.

## Supplementary material

For supplementary material accompanying this paper visit http://doi.org/10.1192/j.eurpsy.2023.10. click here to view supplementary material
