# Supplementary material for: Association between impaired glucose metabolism and long-term prognosis at the time of diagnosis of depression: Impaired glucose metabolism as a promising biomarker proposed through a machine-learning approach
Source: Eur Psychiatry. 2023 Feb 3;66(1):e21. doi: 10.1192/j.eurpsy.2023.10 (PMC9970146; doi:10.1192/j.eurpsy.2023.10)
Supplement: Supplementary file 1 [file S092493382300010Xsup001.docx]

**Supplementary Materials**

**Method S1. Data source**

**Ajou University School of Medicine (AUSOM)**

The Ajou University School of Medicine (AUSOM) database is the EHR database of 3 398 600 patients who visited The Ajou University School of Medicine in South Korea from 1994 to 2021. The AUSOM database is in the form of the OMOP-CDM version 5.3.

**Daegu Catholic Medical Center (DCMC)**

The Daegu Catholic Medical Center (DCMC) database is the EHR database of 906 587 patients who visited the the Daegu Catholic Medical Center in South Korea from 2005 to 2021. The DCMC database is also in the form of the OMOP-CDM version 5.3.

**Wonkwang University Hospital (WKUH)**

The Wonkwang University Hospital (WKUH) database is the EHR database of the Wonkwang University Hospital in South Korea and has data from 837 461 patients who visited the Wonkwang University Hospital from 1998 to 2021. The WKUH database was also included in the OMOP-CDM version 5.3.

**Kyung Hee University Hospital at Gangdong (KHNMC)**

The Kyung Hee University Hospital at Gangdong (KHNMC) database is the EHR database of the Kyung Hee University Hospital in South Korea and has data from 805 332 patients who visited the hospital from 2006 to 2021. The KHNMC database was also included in the OMOP-CDM version 5.3.

**Method S2. Cohort definition**

**1. Patients who were having a new depressive episode**

Initial Event Cohort

People having any of the following:

• condition occurrence of depression for the first time in the person’s history

with continuous observation of at least 365 days prior and 0 days after event index date and limit initial events to: earliest event per person.

Restrict initial events to

Having any of the following criteria:

Inclusion Criteria #1: with condition occurrence of depression

• With at least 1 condition occurrences of ‘depression’, starting between 1 days after and all days after cohort entry start date.

Inclusion Criteria #2: with drug exposure of antidepressant medication

• With at least 2 occurrences of a drug exposure of ‘antidepressants’, starting between 0 days before and all days after cohort entry start date.

Inclusion Criteria #3: with history of psychiatric procedure

• With at least 2 occurrences of a procedure occurrence of ‘psychiatric procedure’, starting between 0 days before and all days after cohort entry start date.

Having all of the following criteria:

Inclusion Criteria #4: without history of diabetes

• With at least 0 condition occurrences of ‘diabetes’, starting between all days before and 0 days before cohort entry start date.

Inclusion Criteria #5: without history of complication due to diabetes

• With at least 0 condition occurrences of ‘complications due to diabetes’, starting between all days after and 0 days before cohort entry start date.

Inclusion Criteria #6: without drug exposure of drugs for diabetes

• With at least 0 occurrences of a drug exposure of ‘drugs for diabetes’, starting between all days before and 0 days before cohort entry start date.

Inclusion Criteria #7: without schizophrenia and bipolar disorder

• With at least 0 occurrences of a drug exposure of ‘schizophrenia spectrum and bipolar disorder’, starting between all days before and 0 days before cohort entry start date.

Inclusion Criteria #8: without prior history of psychosis

• With at least 0 occurrences of a drug exposure of ‘psychosis’, starting between all days before and 1 days before cohort entry start date.

Inclusion Criteria #9: with laboratory test for IGM for 1-year follow up

• With at least 1 occurrences of a measurement of 'fasting blood glucose or HBA1c’, starting between 0 days after and 365 days after cohort entry start date.

Limit qualifying cohort to earliest event per person.

End Date Strategy

No end date strategy selected. By default, the cohort end date will be the end of the observation period that contains the index event.

Cohort Collapse Strategy:

Collapse cohort by era with a gap size of 0 days.

**2. Impaired glucose metabolism**

Initial Event Cohort

People having any of the following:

• a measurement of 'fasting blood glucose' with value as number greater or equal to 100

• a measurement of 'HbA1c' with value as number greater or equal to 5.7

with continuous observation of at least 0 days prior and 0 days after event index date and limit initial events to: earliest events per person.

Limit qualifying cohort to all events per person.

End Date Strategy

Event will persist until: fixed duration (0 days) relative to initial event

Cohort Collapse Strategy: Collapse cohort by era with a gap size of 0 days.

**3. Risk of hospitalization for depression**

Initial Event Cohort

People having any of the following:

• a visit occurrence of ‘Inpatient Visit’ or ‘Emergency room and Inpatient visit’

with continuous observation of at least 0 days prior and 0 days after event index date and limit initial events to: all events per person.

Restrict initial events to

Having all of the following criteria:

Inclusion Criteria #1: with a condition occurrence of depression

• With at least 1 occurrences of a condition occurrence of ‘depression’, starting between 0 day before and 0 day after cohort entry start date.

Inclusion Criteria #2: with a procedure occurrence of psychiatric procedure

• With at least 1 occurrences of a procedure occurrence of ‘psychiatric procedure’, starting between 0 day before and 14 days after cohort entry start date.

Inclusion Criteria #3: washout period for hospitalization

• With at least 0 occurrence of a visit occurrence of ‘Inpatient Visit’ or ‘Emergency room and Inpatient visit’, starting between 14 days before and 1 day before cohort entry start date.

Limit qualifying cohort to all events per person.

End Date Strategy

Event will persist until: fixed duration (0 days) relative to initial event

Cohort Collapse Strategy: Collapse cohort by era with a gap size of 0 days.

**Method S3. Code list for definitions**

1. **Depression**

| OMOP Concept Id | OMOP Concept Name | Domain | Vocabulary | Excluded | Descendants | Mapped |
| --- | --- | --- | --- | --- | --- | --- |
| 440383 | Depressive disorder | Condition | SNOMED | NO | YES | NO |
| 442306 | Adjustment disorder with depressed mood | Condition | SNOMED | NO | YES | NO |
| 4175329 | Organic mood disorder of depressed type | Condition | SNOMED | NO | YES | NO |
| 436665 | Bipolar disorder | Condition | SNOMED | YES | YES | NO |

1. **Antidepressants**

| OMOP Concept Id | OMOP Concept Name | Domain | Vocabulary | Excluded | Descendants | Mapped |
| --- | --- | --- | --- | --- | --- | --- |
| 21604686 | ANTIDEPRESSANTS | Drug | ATC | NO | YES | NO |

1. **Psychiatric procedure**

| OMOP Concept Id | OMOP Concept Name | Domain | Vocabulary | Excluded | Descendants | Mapped |
| --- | --- | --- | --- | --- | --- | --- |
| 45887951 | Psychotherapy Services and Procedures | Procedure | CPT4 | NO | YES | NO |
| 4327941 | Psychotherapy | Procedure | SNOMED | NO | YES | NO |
| 2795675 | Mental Health, Individual Psychotherapy | Procedure | ICD10PCS | NO | YES | NO |
| 2795842 | Mental Health, Electroconvulsive Therapy | Procedure | ICD10PCS | NO | YES | NO |
| 4030840 | Electroconvulsive therapy | Procedure | SNOMED | NO | YES | NO |

1. **Diabetes**

| OMOP Concept Id | OMOP Concept Name | Domain | Vocabulary | Excluded | Descendants | Mapped |
| --- | --- | --- | --- | --- | --- | --- |
| 201820 | Diabetes mellitus | Condition | SNOMED | NO | YES | NO |

1. **Complication due to diabetes**

| OMOP Concept Id | OMOP Concept Name | Domain | Vocabulary | Excluded | Descendants | Mapped |
| --- | --- | --- | --- | --- | --- | --- |
| 442793 | Complication due to diabetes mellitus | Condition | SNOMED | NO | YES | NO |

1. **Drugs for diabetes**

| OMOP Concept Id | OMOP Concept Name | Domain | Vocabulary | Excluded | Descendants | Mapped |
| --- | --- | --- | --- | --- | --- | --- |
| 21600712 | DRUGS USED IN DIABETES | Drug | ATC | NO | YES | NO |

1. **Fasting blood glucose**

| OMOP Concept Id | OMOP Concept Name | Domain | Vocabulary | Excluded | Descendants | Mapped |
| --- | --- | --- | --- | --- | --- | --- |
| 3037110 | Fasting glucose [Mass/volume] in Serum or Plasma | Measurement | LOINC | NO | NO | NO |
| 3035250 | Fasting glucose [Mass/volume] in Capillary blood by Glucometer | Measurement | LOINC | NO | NO | NO |
| 3040820 | Glucose [Mass/volume] in Serum or Plasma --pre-meal | Measurement | LOINC | NO | NO | NO |

1. **HbA1c**

| OMOP Concept Id | OMOP Concept Name | Domain | Vocabulary | Excluded | Descendants | Mapped |
| --- | --- | --- | --- | --- | --- | --- |
| 3004410 | Hemoglobin A1c/Hemoglobin.total in Blood | Measurement | LOINC | NO | YES | NO |

1. **Visit**

| OMOP Concept Id | OMOP Concept Name | Domain | Vocabulary | Excluded | Descendants | Mapped |
| --- | --- | --- | --- | --- | --- | --- |
| 262 | Emergency Room and Inpatient Visit | Visit | Visit | NO | YES | NO |
| 9201 | Inpatient Visit | Visit | Visit | NO | YES | NO |

1. **Schizophrenia spectrum and bipolar disorder**

| OMOP Concept Id | OMOP Concept Name | Domain | Vocabulary | Excluded | Descendants | Mapped |
| --- | --- | --- | --- | --- | --- | --- |
| 435783 | Schizophrenia | Condition | SNOMED | NO | YES | NO |
| 4286201 | Schizoaffective disorder | Condition | SNOMED | NO | YES | NO |
| 432590 | Delusional disorder | Condition | SNOMED | NO | YES | NO |
| 434010 | Schizotypal personality disorder | Condition | SNOMED | NO | YES | NO |
| 35207135 | Shared psychotic disorder | Condition | ICD-10-CM | NO | YES | NO |
| 37117049 | Substance induced psychotic disorder | Condition | SNOMED | NO | YES | NO |
| 4335169 | Acute transient psychotic disorder | Condition | SNOMED | NO | YES | NO |
| 436665 | Bipolar disorder | Condition | SNOMED | NO | YES | NO |

1. **Psychosis**

| OMOP Concept Id | OMOP Concept Name | Domain | Vocabulary | Excluded | Descendants | Mapped |
| --- | --- | --- | --- | --- | --- | --- |
| 436073 | Psychotic disorder | Condition | SNOMED | NO | YES | NO |

**Table S1. Baseline characteristics for study population with or without IGM in DCMC**

| **Variable** | | **Without IGM**  **(n = 1,941)** | | **With IGM**  **(n = 188)** | $\boldsymbol{\chi}_{\mathbf{(}\boldsymbol{df}\mathbf{)}}^{\mathbf{2}}$ | | **P-value** | | |
| --- | --- | --- | --- | --- | --- | --- | --- | --- | --- |
| **Age group, n (%)** |  | |  | | | 7.99(3) | | 0.04 |  |
| < 20 | | 54 (2.8) | | 2 (1.0) |  | |  | | |
| 20 – 39 | | 157 (8.1) | | 3 (1.5) |  | |  | | |
| 40 – 59 | | 598 (30.8) | | 45 (23.9) |  | |  | | |
| ≥ 60 | | 1,132 (58.3) | | 138 (73.4) |  | |  | | |
| **Sex, n (%)** | | | | | | | | |  |
| Male | | 572 (29.5) | | 50 (26.6) | 0.55(1) | | 0.45 | | |
| **Medical history, n (%)** | | | | | | | | |  |
| Chronic liver disease | | 47 (2.4) | | 2 (1.1) | 0.86(1) | | 0.35 | | |
| Renal impairment | | 31 (1.6) | | 4 (2.1) | 0.06(1) | | 0.81 | | |
| Hyperlipidemia | | 479 (24.7) | | 37 (19.7) | 2.06(1) | | 0.15 | | |
| Obesity | | 10 (0.5) | | 2 (1.1) | 0.20(1) | | 0.65 | | |
| Hypertension | | 481 (24.8) | | 44 (23.4) | 0.11(1) | | 0.74 | | |
| Rheumatoid arthritis | | 81 (4.2) | | 5 (2.6) | 0.66(1) | | 0.42 | | |
| **Psychiatric history, n (%)** | | | | | | | | |  |
| Anxiety disorder | | 400 (20.6) | | 31 (16.5) | 1.55(1) | | 0.21 | | |
| Sleep disorder | | 167 (8.6) | | 10 (5.3) | 2.01(1) | | 0.16 | | |
| Neurodevelopmental disorder | | 20 (1.0) | | 0 (0.0) | 1.01(1) | | 0.32 | | |

**Table S2. Baseline characteristics for study population with or without IGM in WKUH**

| **Variable** | | **Without IGM**  **(n = 2,390)** | | **With IGM**  **(n = 327)** | $\boldsymbol{\chi}_{\mathbf{(}\boldsymbol{df}\mathbf{)}}^{\mathbf{2}}$ | | **P-value** | | |
| --- | --- | --- | --- | --- | --- | --- | --- | --- | --- |
| **Age group, n (%)** |  | |  | | | 2.25(3) | | 0.52 |  |
| < 20 | | 62 (2.6) | | 6 (1.8) |  | |  | | |
| 20 – 39 | | 280 (11.7) | | 23 (7.0) |  | |  | | |
| 40 – 59 | | 865 (36.2) | | 107 (32.7) |  | |  | | |
| ≥ 60 | | 1,183 (49.5) | | 191 (58.5) |  | |  | | |
| **Sex, n (%)** | | | | | | | | |  |
| Male | | 887 (37.1) | | 135 (41.4) | 1.96(1) | | 0.16 | | |
| **Medical history, n (%)** | | | | | | | | |  |
| Chronic liver disease | | 40 (1.7) | | 7 (2.1) | 0.15(1) | | 0.70 | | |
| Renal impairment | | 48 (2.0) | | 10 (3.0) | 1.05(1) | | 0.30 | | |
| Hyperlipidemia | | 342 (14.3) | | 57 (17.5) | 1.99(1) | | 0.16 | | |
| Obesity | | 1 (0.4) | | 0 (0.0) | 0.01(1) | | 1.00 | | |
| Hypertension | | 659 (27.6) | | 108 (33.2) | 3.96(1) | | 0.05 | | |
| Rheumatoid arthritis | | 17 (0.7) | | 6 (1.8) | 3.09(1) | | 0.07 | | |
| **Psychiatric history, n (%)** | | | | | | | | |  |
| Anxiety disorder | | 320 (13.4) | | 22 (6.6) | 11.00(1) | | <0.01 | | |
| Sleep disorder | | 272 (11.4) | | 29 (8.8) | 1.59(1) | | 0.21 | | |
| Neurodevelopmental disorder | | 17 (0.7) | | 0 (0.0) | 1.33(1) | | 0.24 | | |

**Table S3. Baseline characteristics for study population with or without IGM in KHNMC**

| **Variable** | | **Without IGM**  **(n = 842)** | | **With IGM**  **(n = 28)** | $\boldsymbol{\chi}_{\mathbf{(}\boldsymbol{df}\mathbf{)}}^{\mathbf{2}}$ | | **P-value** | | |
| --- | --- | --- | --- | --- | --- | --- | --- | --- | --- |
| **Age group, n (%)** |  | |  | | | 7.77(3) | | 0.05 |  |
| < 20 | | 33 (3.9) | | 0 (0.0) |  | |  | | |
| 20 – 39 | | 156 (18.5) | | 4 (14.3) |  | |  | | |
| 40 – 59 | | 332 (39.4) | | 9 (32.1) |  | |  | | |
| ≥ 60 | | 321 (38.2) | | 15 (53.6) |  | |  | | |
| **Sex, n (%)** | | | | | | | | |  |
| Male | | 214 (25.4) | | 9 (32.1) | 0.34(1) | | 0.56 | | |
| **Medical history, n (%)** | | | | | | | | |  |
| Chronic liver disease | | 3 (0.4) | | 1 (3.6) | 1.11(1) | | 0.29 | | |
| Renal impairment | | 17 (2.0) | | 2 (7.1) | 1.36(1) | | 0.24 | | |
| Hyperlipidemia | | 9 (1.1) | | 1 (3.6) | 0.10(1) | | 0.75 | | |
| Obesity | | 4 (0.5) | | 3 (10.7) | 23.9(1) | | <0.01 | | |
| Hypertension | | 110 (13.1) | | 9 (32.1) | 6.82(1) | | <0.01 | | |
| Rheumatoid arthritis | | 7 (0.8) | | 0 (0.0) | 0.01(1) | | 1.00 | | |
| **Psychiatric history, n (%)** | | | | | | | | |  |
| Anxiety disorder | | 290 (34.5) | | 14 (50.0) | 2.24(1) | | 0.13 | | |
| Sleep disorder | | 31 (3.7) | | 0 (0.0) | 0.27(1) | | 0.61 | | |
| Neurodevelopmental disorder | | 8 (1.0) | | 0 (0.0) | 0.01(1) | | 1.00 | | |

**Table S4. Performance of machine learning models in predicting IGM**

| **Performance metrics** | **LR with LASSO** | **XGBoost** | **Random forest** |
| --- | --- | --- | --- |
| Accuracy | 0.667 | 0.615 | 0.539 |
| AUROC | 0.781 | 0.739 | 0.730 |
| AUPRC | 0.338 | 0.260 | 0.297 |

AUROC, area under the receiver operating characteristic curve; AUPRC, area under the precision-recall curve; LR, logistic regression; LASSO, least absolute shrinkage and selection operator; XGBoost, extreme gradient boosting.

**Table S5. The positive or negative contribution to the risk of predicting IGM among top 10 important predictors**

| **Rank** | **LR with LASSO** | **XGBoost** | **Random forest** |
| --- | --- | --- | --- |
| **1** | Normal range of blood glucose level within one year before diagnosis | Normal range of blood glucose level within one year before diagnosis | Normal range of blood glucose level within one year before diagnosis |
| **2** | Normal range of blood calcium level within one year before diagnosis | Measurement of blood aspartate aminotransferase level within one year before diagnosis | Measurement of blood alanine aminotransferase level within one year before diagnosis |
| **3** | Measurement of urinalysis within one month before diagnosis | Measurement of blood triglyceride within one year before diagnosis | Measurement of blood cholesterol level within one year before diagnosis |
| **4** | Measurement of blood platelet within one year before diagnosis | NSAIDs prescription within one month before diagnosis | Measurement of blood alanine aminotransferase level within one year before diagnosis |
| **5** | CT within one month before diagnosis | Measurement of urinalysis within one year before diagnosis | Measurement of blood aspartate aminotransferase level within one year before diagnosis |
| **6** | Measurement of C reactive protein within one month before diagnosis | Antipsychotics prescription within one year before diagnosis | Normal range of blood bilirubin level within one year before diagnosis |
| **7** | Drugs for peptic ulcer prescription at diagnosis | SSRI prescription at diagnosis | Measurement of uric acid level within one year before diagnosis |
| **8** | Measurement of blood cholesterol level within one year before diagnosis | HMG CoA reductase inhibitors prescription within one month before diagnosis | Measurement of creatinine level within one year before diagnosis |
| **9** | Antipsychotics prescription within one year before diagnosis | Measurement of blood hepatitis B virus test within one month before diagnosis | Measurement of blood albumin level within one year before diagnosis |
| **10** | Normal range of blood aspartate aminotransferase level within one year before diagnosis | Haloperidol prescription at diagnosis | Measurement of blood protein level within one year before diagnosis |

*Notes*: Red areas indicate an increase in the risk of predicting IGM, and blue areas indicate a decrease in the risk of predicting IGM; NSAID, Non-steroidal anti-inflammatory drugs; CT, Computed tomography; SSRI, Selective serotonin reuptake inhibitor; LR, logistic regression; LASSO, least absolute shrinkage and selection operator; XGBoost, extreme gradient boosting.
